# Involvement of voltage-dependent anion channel (VDAC) in dengue infection

**DOI:** 10.1038/srep35753

**Published:** 2016-10-25

**Authors:** Kunlakanya Jitobaom, Natthida Tongluan, Duncan R. Smith

**Affiliations:** 1 Institute of Molecular Biosciences Mahidol University, Salaya Campus, 25/25 Phuttamonthon Sai 4, Salaya, Nakhon Pathom 73170, Thailand; 2 Center for Emerging and Neglected Infectious Diseases, Mahidol University, Salaya Campus, 25/25 Phuttamonthon Sai 4, Salaya, Nakhon Pathom 73170, Thailand

## Abstract

During infection, dengue virus (DENV) proteins interact with host cellular constituents promoting the remodeling of the cell to facilitate virus production. While a number of interacting proteins have been identified for DENV non-structural proteins, far fewer interacting partners have been identified for the DENV structural proteins. One protein that has been identified as a DENV E protein interacting protein is the cellular chaperone GRP78. GRP78 has been shown to have a number of cellular interacting partners including the voltage-dependent anion channel (VDAC). In this study we confirmed the interactions between GRP78 and DENV E protein and between GRP78 and VDAC. VDAC was shown to be re-localized during DENV infection, with no change in levels of protein expression. VDAC is predominantly located on the outer membrane of mitochondria and our result is consistent with movement of the mitochondria towards the ER during DENV infection. Down regulation of VDAC through siRNA significantly reduced DENV protein expression, as well as the percentage infection and output virus titer. Our results suggest that VDAC plays an important role in DENV infection.

Despite the recent introduction of a vaccine^1^ in a few countries, infections with the mosquito transmitted dengue virus (DENV) remain a significant worldwide public health problem. It has been estimated that nearly 400 million new infections occur each year, of which approximately one-quarter are symptomatic to some degree^2^. Where DENV infection is symptomatic there is a broad spectrum of presentation ranging from mild flu-like symptoms to a severe life threatening syndrome characterized by significant plasma leakage termed dengue shock syndrome^3^. In the absence of a specific therapeutic drug, treatment of DENV infections is mainly supportive with management of specific symptoms.

There are four distinct DENVs, namely DENV 1 to 4^4^, which are closely related but antigenically distinct. The DENV virion is composed of three proteins (capsid (C), envelope (E) and membrane (M)) with a lipid envelope, and the genetic material is a positive sense single stranded RNA molecule of approximately 11 kb^4^. The DENV genome contains one open reading frame encoding for the three structural proteins as well as the seven non-structural proteins (NS1, NS2A, NS2B, NS3, NS4A, NS4B and NS5) which direct viral replication^5^,^6^.

Infection of a permissive host cell begins with the binding of the DENV E protein to a host cell receptor protein, and a number of such proteins have been identified (reviewed in Cruz-Oliveira, C. *et al*.^7^). Virus internalization occurs predominantly by clathrin-mediated endocytosis^8^,^9^ followed by membrane fusion and release of the nucleocapsid into cytoplasm. The viral genome is translated into viral structural and non-structural proteins, which mediate viral genome replication and new progeny virus assembly and egress from the cell^5^.

During DENV replication the host cellular processes are manipulated to create a favorable environment for viral replication and assembly^10^. This is achieved through a number of mechanisms, but particularly through the direct interaction of viral proteins with host cellular proteins to modulate their expression or activity^11^,^12^. This presupposes that viral protein possess other functions in addition to a direct role in viral replication or viral assembly. For example, in addition to being the viral polymerase and methyltransferase, DENV NS5 protein inhibits the cellular anti-viral IFN response by binding STAT2 and promoting its ubiquitination and subsequent proteasome mediated degradation^13^.

The DENV E protein is the viral receptor binding protein, and as noted earlier a number of receptor proteins have been identified^7^. Interestingly however, only a few cytoplasmic interacting partners of DENV E protein have been identified. Cytoplasmic proteins that have been identified to interact with DENV E protein include actin^14^,^15^ as well as GRP78, calreticulin and calnexin^16^.

GRP78 is multifunctional protein^17^, mainly localized in ER where it functions as an ER resident chaperone and component of unfolded protein response^18^. Previous studies have documented the involvement of GRP78 in DENV replication, and interactions between GRP78 and DENV E protein at a number of stages of the DENV replication cycle have been proposed^16^,^19^,^20^. Several studies have reported cell surface localization of GRP78 and a number of interacting partners including the major histocompatibility complex class I, tetracarcinoma-derived growth factor 1 (Cripto 1) and the voltage dependent anion channel (VDAC)^21^ have been identified.

VDAC is mainly localized in the outer membrane of mitochondria, controlling metabolites transferring between mitochondria and the other cell compartments^22^,^23^. VDAC is also found on the cell surface, but its function in this cellular compartment is unclear^24^. There are three isoforms of VDAC (VDAC1, VDAC2 and VDAC3) in mammalian cells^25^, but studies have shown that they are not equally abundant, with VDAC1 being 10 times more abundant than VDAC2 and 100 times more abundant than VDAC3^26^. Thus the majority of VDAC expressed inside cells is VDAC1.

In a recent study we observed an interaction between Japanese encephalitis virus (JEV) E protein and GRP78 as well as the interaction between GRP78 and voltage dependent anion channel (VDAC) in insect (C6/36) cells^27^. Neither GRP78 nor VDAC were shown to play a role in JEV internalization to insect cells, and while there was no direct interaction between JEV E protein and VDAC, we observed significant relocalization of VDAC during JEV infection^27^. This study sought to determine whether VDAC played a role in DENV infection of mammalian cells, which might imply a broadly conserved mechanism in flavivirus infections. In addition the role of VDAC in DENV infection was assessed through siRNA knockdown of VDAC expression to determine if VDAC plays a functional role in DENV infection.

## Results

### The interaction between DENV E protein and GRP78

**Figure 1:**
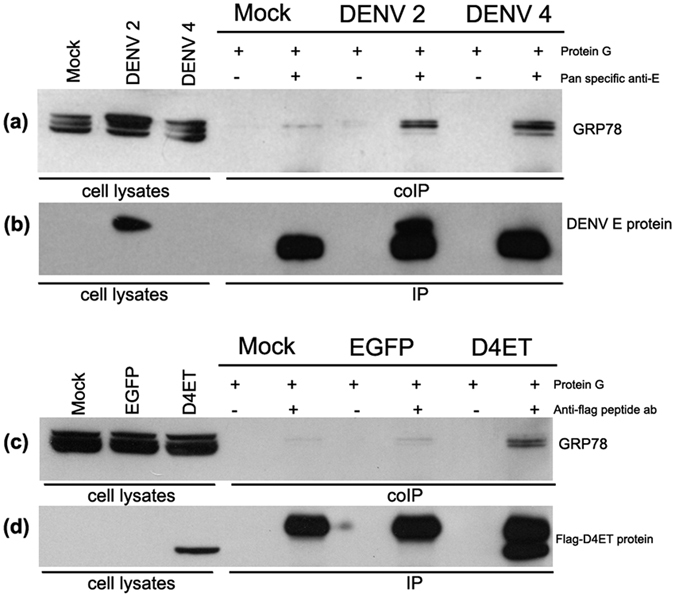
Interaction between DENV E protein and GRP78. HEK293T/17 cells were mock infected or infected with DENV 2 or DENV 4, and cell lysates were prepared on 2 d.p.i. (**a**) Co-immunoprecipitation assays were performed using a pan specific anti-dengue E protein antibody and western blot analysis was performed using an anti-GRP78 antibody. (**b**) The same membrane was probed with a different pan specific anti-dengue E antibody to confirm E protein immunoprecipitation. (**c**) HEK293T/17 cells were mock transfected or transfected with eukaryotic expression plasmids harboring the sequences of flag peptide-tagged truncated DENV 4 E protein (Flag_D4ET) or EGFP as a transfection control, cells were collected at 2 days post transfection. Co-immunoprecipitation assays were performed using an antibody against the flag peptide and western blot analysis was performed using an anti-GRP78 antibody. (**d**) The same membrane was probed with an antibody against the flag peptide to confirm the immunoprecipitation.

Previous studies have shown an interaction between GRP78 and DENV 2 E protein^16^,^28^. To determine if this interaction occurs with other DENV E proteins, we initially re-validated our optimized infection protocol of HEK293T/17 with DENV 2 and DENV 4 as previously reported^14^. HEK293T/17 cells were therefore either mock infected or infected with DENV 2 and 4 and the percentage infection determined by flow cytometry on days 1 to 3 p.i. While some difference was observed in percent infection on day 1 post infection, infection rates were approximately 80% on day 2 p.i (Supplemental Figure S1) consistent with our previous study^14^. HEK293T/17 cells were therefore again infected with DENV 2 and 4 and on day 2 p.i. DENV E protein was pulled down with a pan specific anti-dengue E protein antibody (MAB8705). The pulled down proteins were separated by electrophoresis and after transfer to solid matrix, membranes were probed with an anti-GRP78 antibody. Results (Fig. 1) showed that GRP78 interacted with both DENV 2 and 4 E proteins. The filter was stripped and re-probed with an anti-DENV E protein antibody, and, as shown in Fig. 1 only DENV 2 E protein was shown to have been pulled down. This is consistent with our previous report which also showed failure of the pan-specific anti-DENV E protein to detect DENV 4 E protein in standard western blots^14^.

To address this point, HEK293T/17 cells were transfected with a previously described^14^ eukaryotic expression plasmid harboring a flag-tagged truncated DENV 4 E protein (pcDNA-FLAG_D4ET) and the pull down repeated, this time using an anti-flag tag antibody. Results (Fig. 1) showed that the truncated DENV 4 E protein interacted with GRP78. Note that the truncated protein which lacks the DENV E protein transmembrane domain migrates faster than the full length protein.

**Figure 2:**
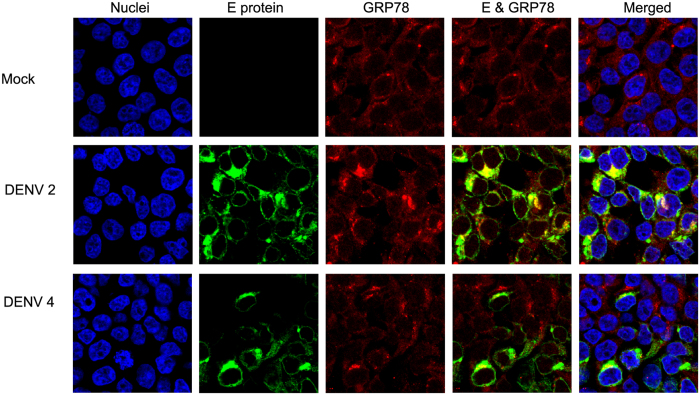
Co-localization between DENV E protein and GRP78. HEK293T/17 cells were mock infected or infected with DENV 2 or DENV 4 for 1 d.p.i. Then the cells were incubated with a pan specific anti-dengue E protein antibody (green) and an anti-GRP78 antibody (red) followed by appropriate secondary antibodies and DAPI (blue). The cells were observed under an Olympus Fluo View 1000 confocal microscope. Representative non-contrast adjusted merged and unmerged images are shown.

Colocalization between DENV 2 E protein and GRP78 has been previously reported^16^,^29^, and to confirm this as well as provide evidence on the possible colocalization between DENV 4 E protein and GRP78, HEK293T/17 cells were mock-infected or infected with DENV 2 or DENV 4 and at 1 d.p.i. cells were collected and examined under a confocal microscope after staining with antibodies against DENV E protein and GRP78. Cells were additionally stained with DAPI for nuclear visualization. As shown in Fig. 2, DENV E protein of both DENV 2 and DENV 4 largely colocalized with GRP78. Predominant expression of DENV E protein was perinuclear. It should be noted that the anti-DENV E protein antibody used showed robust detection of DENV 4 E protein, suggesting that this antibody recognizes an epitope that is lost in some DENVs upon denaturation of the protein (as seen in the western blots; Fig. 1).

### The interaction between GRP78 and VDAC

**Figure 3:**
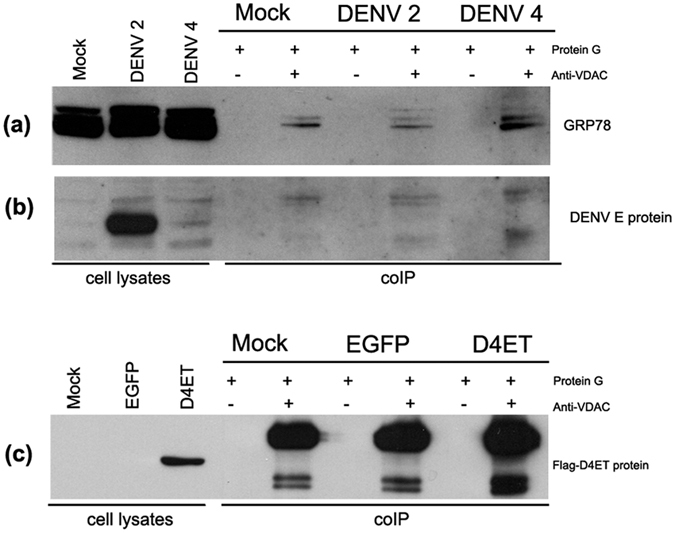
GRP78, but not DENV E protein interacts with VDAC. HEK293T/17 cells were mock infected or infected with DENV 2 or DENV 4, and cell lysates were prepared on 2 d.p.i. Co-immunoprecipitation assays were performed using an anti-VDAC antibody and western blot analysis was undertaken using (**a**) an anti-GRP78 antibody and (**b**) the same membrane was subsequently probed with a pan specific anti-dengue E antibody. (**c**) HEK293T/17 cells were mock transfected or transfected with eukaryotic expression plasmids harboring the sequences of flag peptide-tagged truncated DENV 4 E protein (Flag_D4ET) or EGFP protein as a transfection control, cells were collected at 2 days post transfection. Co-immunoprecipitation assays were performed using an anti-VDAC antibody and western blot analysis was performed using an antibody against the flag peptide.

To investigate the interaction between GRP78 and VDAC, HEK293T/17 cells were mock- infected or infected with DENV 2 or DENV 4, and on day 2 p.i. protein lysates were prepared and VDAC pulled down using an antibody against human VDAC, and the membranes were probed to detect co-immunoprecipitation of GRP78 and DENV E protein. Results (Fig. 3) showed that GRP78 was co-immunoprecipitated with VDAC under all conditions investigated (mock, and DENV 2 and 4 infected). However, re-probing the membrane with and anti-DENV E protein antibody showed that DENV E protein was not pulled down in complex with VDAC (Fig. 3b). However, given that the anti-DENV E protein antibody used is not able to detect denatured DENV 4 E protein, cells were again transfected with pcDNA-FLAG_D4ET, and the co-imunoprecipitation repeated with the membrane being probed with an anti-FLAG antibody. Results confirmed the lack of an interaction between DENV 4 E protein and VDAC (Fig. 3c).

**Figure 4:**
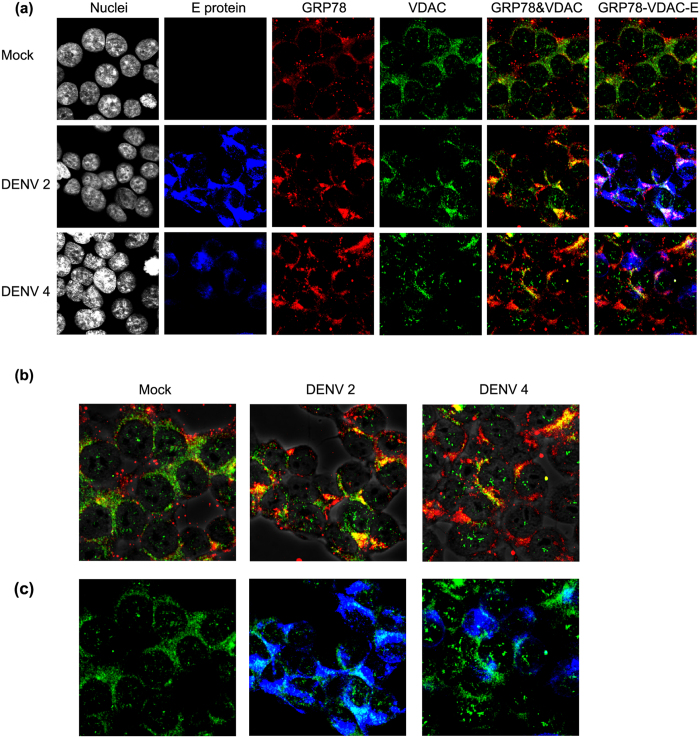
Co-localization between GRP78 and VDAC. (**a**) HEK293T/17 cells were mock infected or infected with DENV 2 or DENV 4 for 1 d.p.i. Then the cells were incubated with pan specific anti-dengue E protein antibody (blue), anti-GRP78 antibody (red) and anti-VDAC antibody (green) followed by appropriate secondary antibodies and DAPI (white). The cells were observed under Olympus Fluo View 1000 confocal microscope. Representative non-contrast adjusted merged and unmerged images are shown. (**b**) Representative non-contrast adjusted merged images of GRP78 and VDAC isolated from (**a**) are shown overlaid with phase-contrast image. (**c**) Isolated representative non-contrast adjusted merged images of VDAC and DENV E protein isolated from (**a**).

Co-localization between GRP78 and VDAC was investigated as described earlier. DENV 2 and DENV 4 infected and mock infected cells were stained with antibodies against DENV E protein, GRP78, and VDAC. As shown in Fig. 4, there was a higher level of co-localization between GRP78 and VDAC in infected cells as compared to mock infected cells. Pearson correlation coefficients (PCC) were determined for each condition. The highest colocalization between GRP78 and VDAC was observed in DENV 2 infected cells, which was significantly greater than that of mock infected cells (PCC ± SD; CIs DENV 2 = 0.51 ± 0.1; ±0.04 and mock = 0.31 ± 0.1; ±0.04, *p* = 0.00). The level of co-localization between GRP78 and VDAC in DENV 4 infected cells was also higher than in mock infected cells although this did not reach statistical significance (PCC ± SD; CIs DENV 4 = 0.38 ± 0.078; ±0.03. *p* = 0.29). VDAC is believed to be predominantly located on the outer membrane of mitochondria^22^,^23^, and to confirm mitochondrial localization HEK293T/17 cells were mock infected or infected with DENV 2 or DENV 4 and at 24 h.p.i. cells were stained with Mito Tracker^®^ Red CMXRos and an antibody directed against VDAC before observation under a confocal microscope. Results confirmed extensive colocalization between VDAC and mitochondria (Supplemental Figure S2).

### Re-localization of VDAC during DENV infection

**Figure 5:**
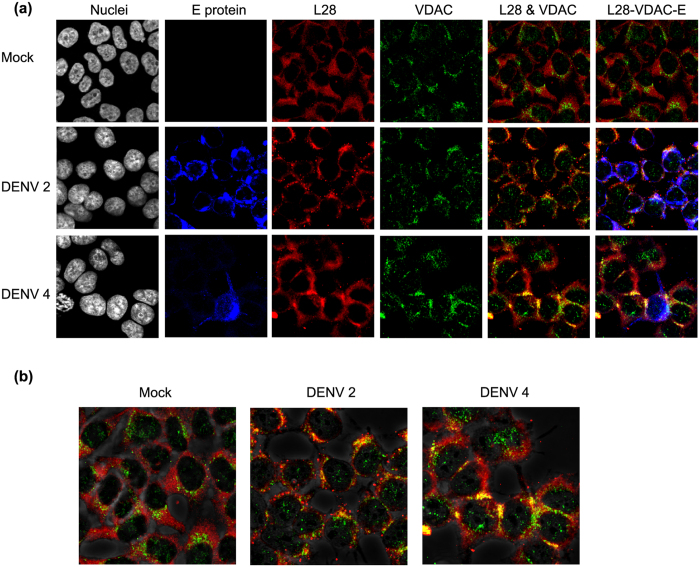
Co-localization between VDAC and ribosomal protein L28. (**a**) HEK293T/17 cells were mock infected or infected with DENV 2 or DENV 4 for 1 d.p.i. and the cells were then incubated with a pan specific anti-dengue E protein antibody (blue), an anti-ribosomal protein L28 antibody (red) and an anti-VDAC antibody (green) followed by appropriate secondary antibodies and DAPI (white). The cells were observed under Olympus Fluo View 1000 confocal microscope. Representative non-contrast adjusted merged and unmerged images are shown. (**b**) Representative non-contrast adjusted merged images of L28 and VDAC are shown overlaid with phase-contrast image.

To determine if there was re-localization of VDAC in response to DENV infection, HEK293T/17 cells were mock-infected or infected with DENV 2 or DENV 4 and at 1 d.p.i. infected cells were collected and stained with antibodies against DENV E protein, GRP78, and ribosomal protein L28 and examined under a confocal microscope. Results (Fig. 5) showed a clear increase in colocalization between VDAC and ribosomal L28 protein in both DENV 2 and DENV 4 infected cells over mock infected cells. Image analysis of colocalization in infected cells was confined to those showing positive signal for E protein and showed that the level of co-localization in DENV 2 and DENV 4 infected cells were significantly increased as compared to colocalization in mock infected cells (PCC ± SD; CIs, Mock = 0.16 ± 0.56; ±0.25, DENV 2 = 0.42 ± 0.1; ±0.05 and DENV 4 = 0.39 ± 0.1; ±0.04, *p* = 0.00). Significantly, while VDAC was observed to predominantly localize in the perinuclear area in mock infected cells, the signal was more diffuse in infected cells (Fig. 5b).

### Regulation of VDAC1 and GRP78 upon DENV infection

**Figure 6:**
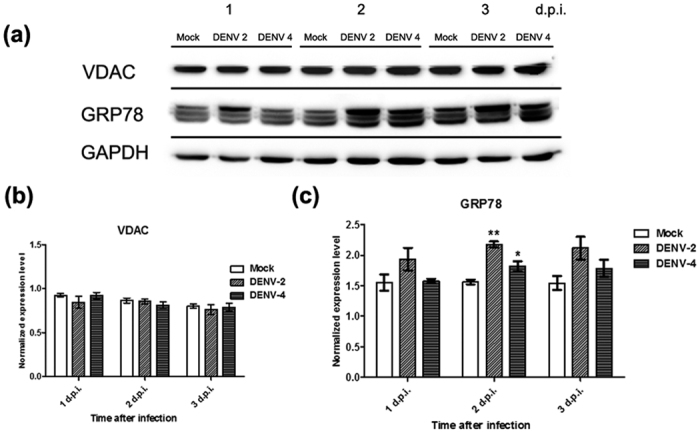
Expression of GRP78 and VDAC during DENV infection. (**a**) HEK293T/17 cells were mock infected or infected with DENV 2 or DENV 4, and lysates prepared on days 1 to 3 p.i. The cell lysates were subjected to SDS-PAGE and western blot analysis using an anti-VDAC antibody, an anti-GRP78 antibody and anti-GAPDH antibody. The experiment was undertaken independently in triplicate. Protein band intensities from (**a**) were quantitated using imageJ image analysis program and analyzed by GraphPad Prism 5 program and the expression of (**b**) VDAC and (**c**) GRP78 were normalized to GAPDH. Error bars show S.E.M.

Studies have previously shown increased expression of GRP78 during DENV infection^16^,^20^,^29^. To confirm this, as well as to investigate the expression of VDAC during infection, HEK293T/17 cells were mock-infected or infected with DENV 2 or DENV 4 and levels of GRP78 and VDAC determined on days 1–3 p.i by western blotting. Results (Fig. 6) showed no significant change of VDAC1 expression in response to DENV infection (Fig. 6a,b), while the levels of GRP78 in DENV 2 or DENV 4 infected cells were significantly increased on 2 d.p.i. as compared with mock infected cells (Fig. 6a,c).

### Effect of knock down of VDAC on DENV infection

To determine whether VDAC has a functional role in DENV infection, expression of VDAC was knocked down using siRNA mediated gene silencing. To optimize silencing of VDAC, HEK293T/17 cells were transfected with one of four siRNAs directed to VDAC (si_hVDAC1–1 to 4). On days 1 to 4 post transfection, VDAC1 expression was investigated by real time PCR and western blot analysis. The results (Supplemental Figure S3) showed that hVDAC1 mRNA expression was reduced significantly by si_hVDAC1–1 transfection at day 1 post transfection. However, the greatest reduction of hVDAC1 mRNA was seen in si_hVDAC1-4 transfected cells on days 2–4 post transfection. From western blot results (Supplemental Figure S3), the greatest and most significant reduction of VDAC1 protein expression was seen in si_hVDAC1-4 transfected cells at days 2 to 4 post transfection. This construct was therefore selected for further use.

To investigate the effects of silencing VDAC on DENV infection, HEK293T/17 cells were mock-transfected or transfected with si_hVDAC1-4 or an irrelevant siRNA (si-GFP) and on day 2 post-transfection cells were infected with DENV 2 or DENV 4. The infected cells were collected at 2 d.p.i. the effect on infection of reduced VDAC expression was investigated by flow cytometry, standard plaque assay and western blot analysis.

**Figure 7:**
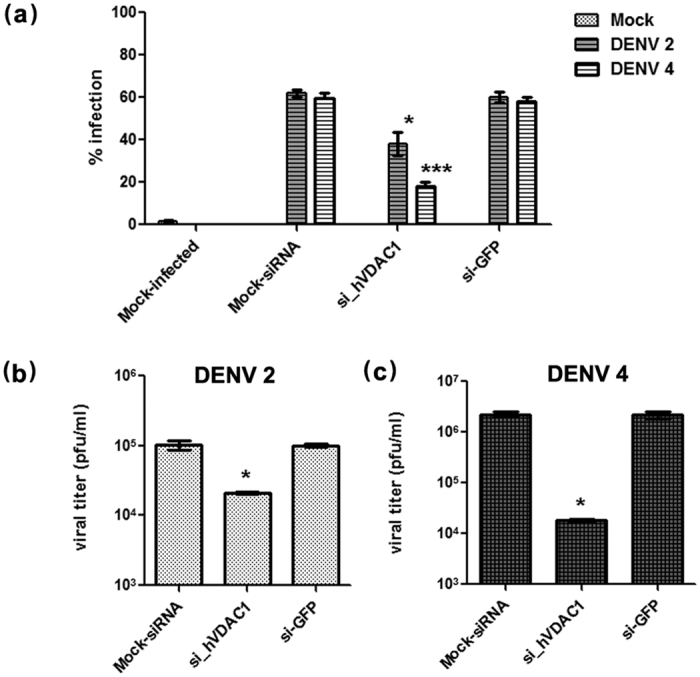
Effect of reduced VDAC expression on infection and virus titer. HEK293T/17 cells were mock transfected or transfected with si_hVDAC1 or si-GFP and subsequently infected with DENV 2 and DENV 4. Mock infected cells were run in parallel. On day 2 p.i. (**a**) percentage infection was determined by flow cytometry and (**b,c**) supernatant was assayed for virus titer by standard plaque assay. Experiment was undertaken independently in triplicate with duplicate plaque assay for (**b**) DENV 2 and (**c**) DENV 4. Error bars show S.E.M.

**Figure 8:**
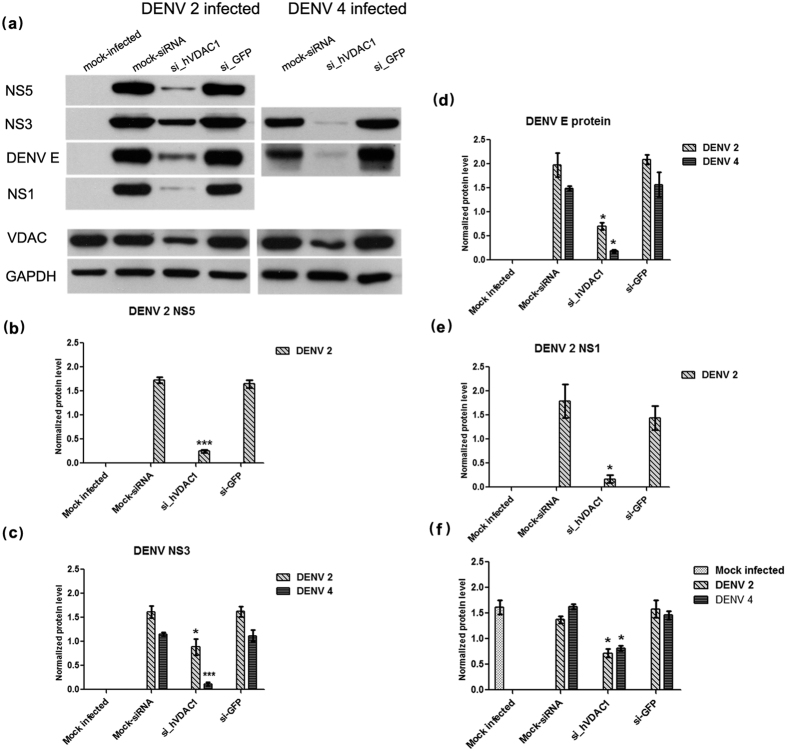
Role of VDAC in DENV infection. (**a**) HEK293T/17 cells were mock transfected or transfected with si_hVDAC1 or si-GFP and subsequently infected with DENV 2 and DENV 4. Mock infected cells were run in parallel. On day 2 p.i. cell lysates were prepared and expression of DENV NS5, NS3, E and NS1 proteins together with VDAC and GAPDH were determined by western blot analysis. Experiments were undertaken independently in triplicate and protein band intensity from were quantitated using the imageJ image analysis program and analyzed by GraphPad Prism 5 progarm. The levels of (**b**) DENV 2 NS5, (**c**) DENV 2 and 4 NS3, (**d**) DENV 2 and 4 E protein, (**e**) DENV 2 NS1, and (**f**) VDAC were normalized to GAPDH. Error bars show S.E.M.

Flow cytometry showed that the percentage infection for both DENV 2 and DENV 4 was significantly reduced in si_hVDAC1-4 transfected and infected cells for both DENV 2 and DENV 4 cells (Fig. 7a), while standard plaque assay showed a significantly reduced virus output for both DENV 2 and DENV 4 (Fig. 7b,c). Western blot analysis confirmed that VDAC expression was specifically and significantly reduced in the si_hVDAC1-4 transfected cells (Fig. 8a,f) and in si_hVDAC1-4 transfected and DENV infected cells, levels of both structural (DENV E protein) and non-structural proteins (NS1, NS3 and NS5) were significantly reduced in both DENV 2 and DENV 4 infections (Fig. 8). Note that the use of a non-reducing loading dye in this experiment for DENV 4 proteins (as opposed to a reducing loading dye used in co-immunoprecipitation experiments and for DENV 2 in this experiment) allowed the detection of DENV 4 E protein using antibody HB112^30^. Despite this however, no signal was detected for DENV 4 NS1 and NS5 proteins (Fig. 8).

## Discussion

GRP78 is a multifunctional chaperone protein^17^. It is primarily resident in the ER where it mediates the induction of the unfolded protein response (UPR) through its interactions with three key proteins, namely IRE1 (Inositol-requiring protein 1), ATF6 (Activating transcription factor 6) and PERK (protein kinase RNA-like endoplasmic reticulum kinase)^18^. Under normal conditions, GRP78 binds and sequesters these proteins, while under conditions of ER stress (such as an influx of unfolded proteins to the ER) GRP78 dissociates from these proteins allowing their subsequent activation^18^. Upon release from GRP78 IRE1 and PERK undergo homodimerization and autophosphorylation leading to increased expression of chaperone proteins and a reduction in translation of cytoplasmic RNAs^31^,^32^, while ATF6 undergoes cleavage in the Golgi compartment, leading to expression of chaperone and other genes^18^,^33^.

An interaction between GRP78 and DENV 2 E protein has been documented previously^16^,^28^, and in this work we have expanded that to show that GRP78 also interacts with DENV 4 E protein. This, coupled with our previous study showing an interaction between JEV E protein and GRP78 in insect cells^27^, suggests that this interaction might be a common interaction in flavivirus infections. In particular these studies raise the question of whether the specific binding of DENV E protein to GRP78 triggers the UPR or whether the interaction is secondary to the mass of unfolded proteins entering the ER during DENV infection. The induction of the UPR in DENV infection has been established to be serotype independent^34^, and as such the observation of a direct interaction between DENV 4 E protein and GRP78 would argue that the specific interaction is an important component of the activation of the UPR. In particular, the UPR is primarily a pro-survival mechanism that seeks to re-establish cellular homeostasis^18^ and thus the triggering of the UPR through a specific interaction may serve to delay the onset of apoptosis, providing a greater period for viral replication.

Studies have shown that apoptosis is a common consequence of DENV infection of mammalian cells and a number of mechanisms including both intrinsic and extrinsic apoptosis pathways have been proposed^29^,^35^,^36^,^37^,^38^,^39^. In intrinsic apoptosis, cytochrome C is released from mitochondria as a consequence of dysregulation of the mitochondrial membrane potential leading to formation of the apoptosome and subsequent induction of apoptosis^40^. Mitochondria are also involved in extrinsic apoptosis as one of the targets of caspase 8, activated through death receptor activation is Bid, and truncated Bid (tBid) causes mitochondrial outer membrane permeabilization, and subsequent cytochrome C release^41^.

Mitochondria are doubled membranes organelles and they have their own genetic material which encodes for 2 rRNAs, 22 tRNAs and 13 polypeptides that are intrinsic protein subunits of the oxidative phosphorylation system and, in addition to their roles in apoptosis, mitochondria are integral to the processes of fatty acid biosynthesis and energy production^42^. Both of these processes are critical to DENV replication. Autophagy, the cellular catabolic process is upregulated in DENV infection^43^,^44^, and it has been proposed that this serves to primarily increase the amount of energy available for DENV replication through increased β-oxidation^45^.

Studies have shown that the cellular consequences of DENV infection are markedly different for mammalian and insect cells. While apoptosis is a common consequence of mammalian cell infection^29^,^35^,^36^,^37^,^38^,^39^, insect cells such as C6/36 cells can be maintained as infected in culture for long periods of time (several months) with little or no cell deficit^46^. While DENV infection induces apoptosis in mammalian but not insect cells remains to be completely elucidated, but studies have suggested that specific up-regulation of the ubiquitin protein Ub3881 in insect cells serves to specifically increase DENV E protein degradation^47^ and reduce virus output. This would imply that DENV E protein is a major determinant of apoptosis induction in mammalian cells.

In a previous study we showed that VDAC was relocalized in response to JEV infection of insect cells^27^. In this study we have shown that VDAC is relocalized in DENV infection in mammalian cells, and this occurred with both the DENV 2 and DENV 4 isolates investigated. The cell line utilized in this study, HEK293T/17 is of a human embryonic kidney origin^48^, and while this cell line may not represent a bona fide target cell line it is capable of supporting high levels of DENV infection as well as being consistently transfectable, and as such is a suitable model cell line. The results suggests that re-localization of VDAC during flavivirus infection may be a common mechanism. VDAC forms pores in the outer mitochondrial membrane, and the status of pore closure or opening depends on its conductance and VDAC allows the entry and exit of selective molecules such as adenine nucleotides^49^, calcium ions^50^ and other metabolites, thus controlling the connections between mitochondria and the rest of the cell^22^. Our results suggest that the relocalization of VDAC might be a mechanism by which nucleotides and metabolites including ATP are bought nearer to the sites of DENV replication. DENV replication is believed to occur in specific replication vesicles probably generated through re-organization of the ER membranes^51^. Consistent with our earlier study^27^ DENV infection resulted in increased colocalization between GRP78 and VDAC, and increased colocalization between VDAC and a ribosome marker (ribosomal protein L28). These results are consistent with mitochondria being bought nearer to the ER/replication sites, rather than GRP78 relocalizing towards VDAC.

Currently, the three dimensional interaction between VDAC, GRP78 and DENV E protein remains largely unknown. It is believed that DENV E protein interacts with the C-terminus of GRP78^21^, and the C-terminus of GRP78 is believed to be the predominant protein interacting domain, while the N-terminus is believed to contain regulatory domains that mediate C-terminal binding^52^. Although the exact E protein domain mediating binding to GRP78 is not known, GRP78 is also known as binding immunoglobulin protein (BiP) and it has been proposed that GRP78 and DENV E protein interact through the immunoglobulin like structure in the DENV E protein that resides in domain III^53^. Our data which showed that the truncated DENV 4 E protein (which contains domain III) interacted with GRP78 would support this proposal. While the binding domains between VDAC and GRP78 remain to be elucidated, our results would suggest that the binding site of VDAC is at a site distinct from that of the DENV E protein as the presence of DENV E protein does not displace the GRP78-VDAC interaction.

Two recent studies have investigated the role of mitochondria in DENV infection^54^,^55^. In the first study Yu and colleagues showed that the DENV NS2B/NS3 protease cleaved two mitofusins which mediate mitochondrial fusion, resulting in disrupted mitochondrial membrane potential^55^. More recently Chatel-Chaix and colleagues showed altered mitochondrial morphology as a consequence of DENV NS4B protein interacting with mitochondrial proteins^54^. Importantly, Chatel-Chaix and colleagues showed elongation of mitochondria and increased contact with the DENV induced replication vesicles at the ER^54^, thus providing a mechanistic explanation for the increased colocalization between VDAC and GRP78 and L28 observed in this study.

Knock down experiments showed that VDAC plays a role in DENV infection, and there were significant reduction in expression of both structural and non-structural proteins, which was reflected by reduced levels of DENV infection and virus output. This might occur as a consequence of required metabolites being sequestered in the mitochondria, and thus not available for supporting viral replication. Collectively the results suggest that further investigation into the role of mitochondria in DENV infection is warranted.

## Materials and Methods

### Cells and viruses

HEK293T/17 (human embryonic kidney) cells were cultivated in Dulbecco’s modified eagle’s medium (DMEM; Gibco Invitrogen, Carlsbad, CA) supplemented with 10% heat-inactivated fetal bovine serum (FBS, Gibco Invitrogen) without antibiotics at 37 °C in an incubator with 5% CO_2_. LLC-MK_2_ (rhesus monkey kidney) cells were cultivated in DMEM (Gibco Invitrogen) supplemented with 5% FBS and 100 units/ml of penicillin and 100 μg/ml of streptomycin at 37 °C in an incubator with 5% CO_2_. C6/36 (*Aedes albopictus*) cells were cultivated in minimum essential medium (MEM; Gibco Invitrogen) supplemented with 10% FBS, 100 units/ml of penicillin and 100 μg/ml of streptomycin (PAA Laboratories, Linz, Austria) at 28 °C. DENV 2 (strain 16681) and DENV 4 (strain 1036), were propagated in C6/36 cells and the viral titer was determined by standard plaque assay in LLC-MK_2_ cells as previously described^46^.

### Virus infection

HEK293T/17 cells were seeded at a density that allowed 70–80% confluency to be reached within 24 hrs. Then the cells were mock-infected or infected with DENV 2 or DENV 4 at a multiplicity of infection (m.o.i.) of 5 and 20, respectively, for 2 hours at 37 °C. Subsequently, the medium containing viruses was removed and replaced with DMEM containing 10% FBS and cells were incubated under standard condition until the times indicated.

### Flow cytometry

Mock-infected and DENV infected cells were fixed with 4% paraformaldehyde. Then the cells were permeabilized and incubated with a 1:150 dilution of pan specific anti-dengue E protein monoclonal antibody HB114^30^, followed by an appropriate dilution of a secondary antibody conjugated with FITC. DENV infection was analyzed on a BD FACalibur cytometer (Becton Dickinson, BD Biosciences, San Jose, CA) using the CellQuest™ software.

### DENV4 E protein eukaryotic expression constructs

A construct of DENV4 E protein lacking the transmembrane domain (D4ET) and with an added flag-tag was cloned into the pcDNA 3.1+ eukaryotic expression vector (Invitrogen, CA), generating the recombinant plasmids pcDNA-FLAG_D4ET as previously described^14^.

### Co-immunoprecipitation assay (coIP)

For coIP assays using infected cells, HEK293T/17 cells were seeded into 100 mm^2^ tissue culture plates at a density that allowed 70–80% confluency to be reached within 24 hrs. After that the cells were mock-infected or infected with DENV 2 or DENV 4 using the standard infection protocol. For coIP assay using transfected cells, HEK293T/17 cells were seeded at a density that allowed approximately 50% confluency to be reached within 24 hrs. Then the cells were mock-transfected or transfected with pcDNA-FLAG_D4ET or pcDNA-EGFP plasmids using the calcium phosphate mediated transfection method and further cultured for 2 days post-transfection.

Infected or transfected cells were collected at the appropriate time points and the cells were washed once with phosphate-buffer saline (PBS) and subsequently resuspended in pre-cooled lysis buffer (20 mM Tris–HCl pH 7.5, 150 mM NaCl, 1 mM EDTA, 1% Triton X-100, 2.5 mM sodium pyrophosphate, 1 mM β-glycerophosphate, 1 mM Na3VO4, 1 mM PMSF) before the cells were lysed by vortexing followed by centrifugation at 16,000g for 5 min at 4 °C. The cell lysates were transferred to new tubes and protein concentrations were determined by the Bradford assay. To pre-clear cell lysates, 1 mg of cell lysates were incubated with Protein G Sepharose 4 Fast Flow beads (GE Healthcare, Buckinghamshire, UK) and rotated for 1 hour at 4 °C. Subsequently, pre-cleared cell lysates from infection experiments were incubated with or without 1 μg of a mouse pan specific anti-dengue E protein antibody (MAB8705, Millipore, MA) or 1 μg of a rabbit anti-VDAC antibody (46615, Cell Signaling Technology, MA), while pre-cleared cell lysates from transfections were incubated with or without 1 μg of a rabbit anti-OctA-Probe (flag peptide) antibody (sc-807; Santa Cruz Biotechnology Inc.). The mixtures were incubated with gentle rocking overnight at 4 °C following which 30 μl of protein G beads slurry was added to the mixtures and incubation with gentle rocking for 4 hours at 4 °C. To remove unbound proteins, the mixtures were centrifuged at 6000 × g for 5 min and the supernatants were discarded followed by washing four times with IP-lysis buffer. To elute the protein complexes, the pellets were resuspended in 30 μl of 3 × SDS sample loading buffer and heated at 100 °C for 5 min. The beads were removed by centrifugation at 14,000 × g for 3 min. The samples were electrophoretically separated on 12% SDS-polyacrylamide gels and subsequently transferred to nitrocellulose membranes (GE Healthcare, Buckinghamshire, UK).

### Western blot analysis

Proteins were electrophoretically separated on 12% SDS polyacrylamide gels and subsequently transferred to nitrocellulose membranes (GE Healthcare, Buckinghamshire, UK). Co-immunoprecipitation experiments and DENV 2 protein western blots used a reducing protein loading buffer as described above, while DENV 4 western blots used a non-denaturing loading dye (Pierce Lane Marker Non-reducing Sample Buffer, ThermoFisher Scientific). The membranes were blocked with 5% skim milk in TBS/0.1% Tween 20 at room temperature. Then the membranes were incubated with a 1:1000 dilution of goat anti-GRP78 polyclonal antibody (sc-1050; Santa Cruz Biotechnology Inc.), a 1:500 dilution of mouse monoclonal anti-dengue serotype1-4 antibody (MA1-27093, Thermo Scientific, MA), a 1:500 dilution of an anti-flavivirus E protein monoclonal antibody (ATCC HB-112), a 1:3000 dilution of rabbit anti-VDAC antibody (46615, Cell Signaling Technology, MA), 1:5000 dilution of mouse anti-dengue type 2 NS5 antibody (MA5-17295, Thermo Scientific, MA), a 1:3000 dilution of rabbit anti-dengue NS3 antibody (PA5-32199, Thermo Scientific, MA) and a 1:3000 dilution of rabbit anti-dengue type 2 NS1 antibody (PA5-32207, Thermo Scientific, MA) for overnight at 4 °C. For glyceraldehyde 3-phosphate dehydrogenase (GAPDH) the membranes were incubated with a 1:5000 dilution of mouse anti-GAPDH monoclonal antibody (sc-32233; Santa Cruz Biotechnology Inc.) for 1 hour at room temperature. After that the membranes were incubated for 2 hours at room temperature with a 1:8000 dilution of a horseradish peroxidase (HRP) conjugated rabbit anti-mouse IgG, a HRP-conjugated rabbit anti-goat IgG or a HRP-conjugated goat anti-rabbit IgG as appropriate. The antigen-antibody complexes were detected using the Clarity™ Western ECL Substrate (Biorad, CA). The membranes were exposed to autoradiography films or observed by a visible western blot imaging system (Azure c400, Azure Biosystems, Inc., Dublin, CA).

### Immunofluorescence assays

The day prior infection, HEK293T/17 cells were seeded on glass coverslips at a density that allowed 40–50% confluence to be reached within 24 hours. Then the cells were mock-infected or infected with DENV 2 or DENV 4 as described previously, and further cultured under standard conditions for the specific time periods, following which the cells were washed once with PBS and fixed with pre-cooled absolute methanol for 20 min. For cells undergoing Mito Tracker^®^ Red CMX ROS staining (Molecular Probes, Invitrogen, OR) cells were washed once with warm DMEM without FBS and then incubated with DMEM containing Mito Tracker^®^ Red CMX ROS (Molecular Probes, Invitrogen, OR) according to manufacturer’s instruction for 30 min at 37 °C. Subsequently, the medium containing Mito Tracker^®^ Red CMX ROS was removed and the cells were washed once with PBS followed by fixation using 4% paraformaldehyde for 20 min. After removal of fixing reagents the cells were permeabilized with 0.3% Triton X-100 in PBS and blocked with 5% BSA in PBS, each for 10 min. Subsequently the cells were incubated with a 1:100 dilution of a mouse pan specific anti-dengue E protein antibody (MAB8705, Millipore, MA), a 1:50 dilution of goat anti-GRP78 (sc-1050; Santa Cruz Biotechnology Inc.), a 1:50 dilution of rabbit anti-VDAC (sc-98708; Santa Cruz Biotechnology Inc.), or a 1:100 dilution of goat anti-ribosomal protein L28 (sc-14151, Santa Cruz Biotechnology Inc.) at 4 °C for overnight. After that, the cells were washed with 0.03% Triton-X 100 in PBS for four times and incubated with appropriate secondary antibodies at room temperature. The secondary antibodies used were a 1:200 dilution of Alexa 488-conjugated donkey anti-mouse IgG antibody, a 1:50 dilution of Alexa 568-congugated donkey anti-goat IgG antibody, a 1:50 dilution of Alexa 647-congugated donkey anti-rabbit IgG antibody (A21202, A11057, A31573, Molecular Probes, Thermo Fisher Scientific) and a 1:500 dilution of 4′, 6-diamidino-2-phynyllindole (DAPI) (Calbiochem, EMD Chemical, Inc.) for nuclear staining. After washing several times, the cells were mounted onto glass slides using Prolong^®^ Gold antifade reagent (Molecular Probes, Thermo Fisher Scientific). The cells were observed under an Olympus Fluo View 1000 confocal microscope (Olympus, Shinjuku-ku, Tokyo, Japan) and images analyzed with Olympus Fluo View Software version 4.0. For co-localization analysis, the Pearson correlation coefficients (PCC) were determined using the ImageJ analysis program^56^ and the PSC co-localization plugin^57^. The results are described as PCC, with standard deviation (SD) and confidence levels (CIs).

### Optimization of siRNA mediated gene silencing of hVDAC1

Four siRNA templates were designed from target sites on human VDAC1 gene (hVDAC1; NCBI accession no. NG 027817_1). The positions of the target sites were 119-139 bp, 286-306 bp, 320–540 bp and 820–840 bp (si-hVDAC1-1 to 4, respectively). The siRNAs were synthesized from DNA oligonucleotides sequence templates using the *Silencer*^®^ siRNA Construction Kit (Ambion) according to the manufacturer’s protocol. To optimize siRNA knockdown of hVDAC1 expression, HEK293T/17 cells were transfected with 250 pmol of siRNA using Lipofectamine™ RNA*i*MAX (Invitrogen, Carlsbad, CA) by reverse transfection according to the manufacturer’s protocol in individual wells of 12-well tissue culture plates. EGFP siRNA (si-GFP), an irrelevant siRNA, was used as a negative control. The transfected cells were further grown under standard condition for 1, 2, 3 and 4 days post transfection at which point the transfected cells were collected and hVDAC1 mRNA expression was investigated by real time PCR, while VDAC1 protein expression was determined by western blot analysis.

### Effects of gene silencing mediated by siRNA of hVDAC1 on DENV infection

HEK293T/17 cells were transfected with siRNAs hVDAC1 and si-GFP using Lipofectamine™ RNA*i*MAX. On day 2 post-transfection, the cells were infected with DENV 2 or DENV 4 under standard conditions, and on day 2 post-infection the cells were collected, the percentage of infection was determined by flow cytometry and protein expression analysis was performed by western blotting. The culture supernatant was additionally collected to determine viral titer using standard plaque assays.

### Real-time PCR

Total RNA was extracted using TRI reagent (Molecular Research Center, Inc., Cincinnati, OH) according to the manufacturer’s instructions and RNA concentrations were determined by spectrophotometry. First strand cDNA was synthesized using ImProm-II™ reverse transcriptase (Promega, Madison, WI) and oligo (dT). The quantification of gene expression using real-time PCR was performed with specific primers; hVDAC1_F: 5′-GTGCTAGGTTACGAGGGCTG-3′, hVDAC1_R: 5′-CCAAACTCTGTCCCGTCATT-3′, and GAPDH as internal control using GAPDH-F: 5′-GAACATCATCCCTGCCTCTAC-3′, GAPDH-R: 5′-CCTGCTTCACCACCTTCTT-3′ primers. The cycle conditions were 95 °C for 3 min, followed by 40 cycles of 95 °C for 10 sec, 60 °C for 30 sec and extension of 72 °C for 20 sec.

### Statistical analysis

Data were analyzed using GraphPad Prism 5 program (GraphPad Software, Inc., CA). The statistical analysis of significant difference was undertaken by independent t-test using SPSS (SPSS Inc.) with *p*-value ≤ 0.05 taken as significant.

## Additional Information

**How to cite this article**: Jitobaom, K. *et al*. Involvement of voltage-dependent anion channel (VDAC) in dengue infection. *Sci. Rep.* **6**, 35753; doi: 10.1038/srep35753 (2016).

## Supplementary Material

Supplementary Information
