# Supplementary material for: Involvement of voltage-dependent anion channel (VDAC) in dengue infection
Source: Sci Rep. 2016 Oct 25;6:35753. doi: 10.1038/srep35753 (PMC5078847; doi:10.1038/srep35753)
Supplement: Supplementary Information [file srep35753-s1.pdf]

Supplementary Figures

Involvement of voltage-dependent anion channel (VDAC) in dengue infection

Kunlakanya Jitobaom[1], Natthida Tongluan[1] and Duncan R. Smith[1,2*]

[1]Institute of Molecular Biosciences and [2]Center for Emerging and Neglected Infectious Diseases, Mahidol University, Salaya Campus, 25/25 Phuttamonthon Sai 4, Salaya, Nakhon Pathom, Thailand 73170

*Correspondence to: Duncan R. Smith, Molecular Pathology Laboratory, Institute of Molecular Biosciences, Mahidol University, Salaya Campus, 25/25 Phuttamonthon Sai 4, Salaya, Nakhon Pathom, Thailand 73170; Phone: 66 (0) 2441-9003 to 7. Fax: 66 (0) 2441-1013. E-mail: duncan_r_smith@hotmail.com, duncan.smi@mahidol.ac.th

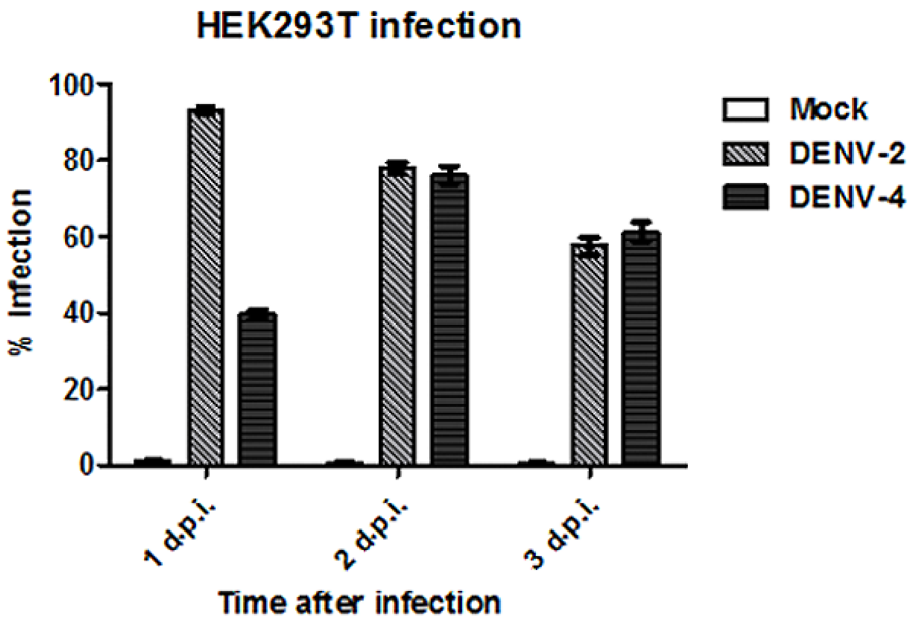

Supplemental Figure S1. Optimization of DENV infection

HEK293T/17 cells were infected with DENV 2 or DENV 4 at m.o.i. 5 or 20, respectively. The percentage of infection was analyzed by flow cytometry. Experiment was undertaken independently in triplicate. Error bars show ± S>E.M.

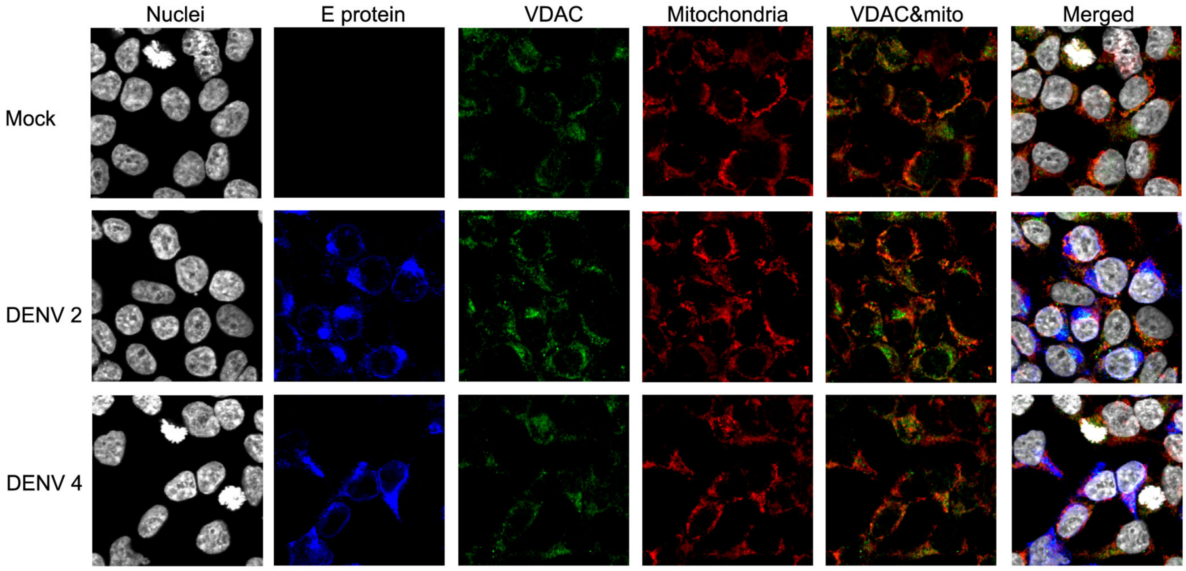

Supplemental Figure S2. Colocalization of VDAC and mitochondria

HEK293T cells were mock-infected or infected with DENV 2 or 4 at m.o.i 5 or 20 respectively. At 24 h.p.i, the cells were stained with Mito Tracker Red CMXRos (red), and antibodies against DENV E protein (blue) and VDAC (green) followed by appropriate secondary antibodies and DAPI for nuclear visualization (white). The cells were observed under an Olympus Fluo View 1000 confocal microscope. Representative, non-contrast adjusted images are shown.

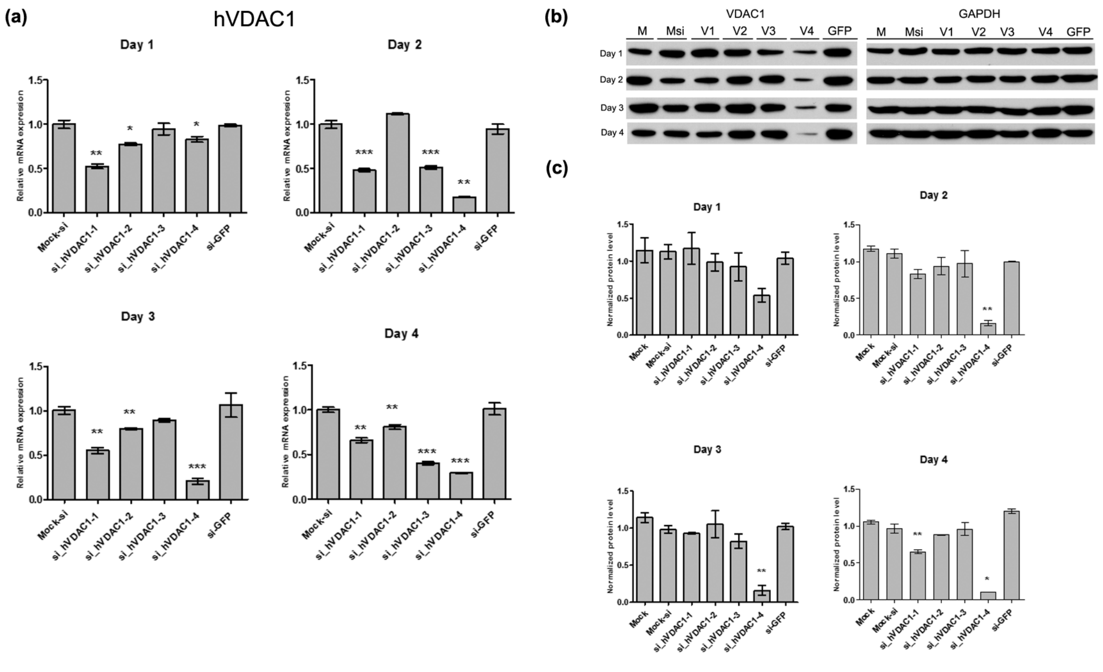

Supplemental Figure S3. Optimization of VDAC knock down by siRNA

HEK293T/17 cells were transfected with one of four siRNAs directed against human VDAC1 (si_hVDAC1-1 to 4), for 1-4 days post transfection. An irrelevant siRNA, si-GFP was used as a negative control. On days 1 to 4 post transfection the transfected cells were collected and the expression of VDAC1 (a) mRNA was investigated by real time PCR and (b) protein was analyzed by western blot analysis. (c) Protein band intensity from (b) were quantitated using imageJ image analysis program and analyzed by GraphPad Prism 5 program. The levels of VDAC1 expression were normalized to GAPDH. Error bars show S.E.M.
